# Complexities and capabilities of Scan4Safety in NHS hospitals: a qualitative study of a national demonstrator site

**DOI:** 10.1136/bmjhci-2024-101366

**Published:** 2026-01-14

**Authors:** Valentina Lichtner, Aleksandra Irnazarow, Stephen Bush, Dawn Dowding, Philip Elphick, Bryony Dean Franklin, Yogini H Jani, Mark Songhurst

**Affiliations:** 1 School of Health Sciences, The University of Manchester Faculty of Biology Medicine and Health, Manchester, UK; 2 Leeds University Business School, University of Leeds, Leeds, UK; 3 Leeds Teaching Hospitals NHS Trust, Leeds, UK; 4 Research Department of Practice and Policy, UCL School of Pharmacy, London, UK; 5 NIHR North West London Patient Safety Research Collaboration, London, UK; 6 Centre for Medicines Optimisation Research and Education, University College London Hospitals NHS Foundation Trust, London, UK

**Keywords:** Electronic Data Processing, Patient Identification Systems, Hospitals

## Abstract

**Objectives:**

Data standards and barcoding technologies are implemented in hospitals to uniquely identify objects, people and locations; streamline the management of supplies and inventories; improve efficiency; reduce waste and improve patient safety and quality of care. This study examined the implementation of the Scan4Safety programme at one NHS demonstrator site to understand the hospital experience of adopting these standards, barcoding and related technologies.

**Methods:**

Exploratory case study design, informed by information infrastructure theory, at one Scan4Safety demonstrator site. Semi-structured interviews were conducted with internal and external stakeholders (n=19), and 67 documents related to the Scan4Safety programme were identified. Interview transcripts and documents underwent thematic analysis.

**Results:**

Key enablers for Scan4Safety included allocated funding, government role/regulation, executive buy-in/wide stakeholder involvement, patient focus, agile/adaptive approach and data linkage. Challenges were both internal and external, mainly pertaining to data quality, work-as-done and trade-offs. Mechanisms of anticipated positive outcomes and potential risks were also identified.

**Discussion:**

Scan4Safety benefits are delivered through tracking and tracing capabilities, and automating data capture, alerts and data linkages. For traceability of devices, the benefits depend on the extent to which items are tracked in inventory and consistent barcode scanning at the point of care.

**Conclusions:**

Linked standards for identification of patients, products, places and procedures, across supplies and hospital processes, constitute a wide-ranging information infrastructure with the potential for significant value to patients and the whole health system.

**WHAT IS ALREADY KNOWN ON THIS TOPIC**

- Data standards encoded in barcodes facilitate tracking and tracing items across health systems, facilitating surveillance and management of resources.
- Little is known about use of barcodes in hospital inventory management, bed management or traceability of implants, especially outside the US context.
- The Scan4Safety programme quantified efficiency savings derived from implementing GS1 standards and barcoding in NHS hospitals in England.

**WHAT THIS STUDY ADDS**

- This study identifies anticipated benefits, as well as trade-offs and new safety risks associated with the use of standards and barcodes for unique identification of patients, beds management and tracking and tracing of medical devices.

**HOW THIS STUDY MIGHT AFFECT RESEARCH, PRACTICE OR POLICY**

- Recommendations are provided to support scaling-up Scan4Safety implementations within a hospital and across the NHS.

## Background

 Patient safety and quality of care in hospital may be improved by the use of barcoding and data standards for the unique identification of objects, people and locations.^1 2^ An example of such standards is those provided by GS1, a global standards organisation operating in healthcare as well as other sectors.^3^ The use of these technologies also contributes to streamlining the management of supplies and inventories, improving efficiency and reducing waste.^1^,^10^ Key patient safety applications or ‘use cases’ of these technologies are medication administration (known as bar coded medication administration; BCMA),^11^ traceability of medical devices for post-market surveillance,^12^,^14^ closed loop chains for blood and transfusion^15^ and tracking samples in pathology.^16^ Centralised inventories and tracing items by unique identifiers are also strategies for resilience when facing supply-chain shortages of medical devices.^17 18^

To support the implementation of GS1 standards and barcoding in NHS hospitals, in 2016–2019, the UK Department of Health & Social Care (DHSC) ran the Scan4Safety demonstrator programme.^19^ The programme funded six English NHS hospital trusts as demonstrator sites, required to adopt GS1 standards for the identification of products (e.g. medical equipment), people (patients) and places (e.g. hospital buildings), recorded in conjunction with processes (e.g. clinical procedures). An audit at the end of the programme identified benefits and lessons learnt across the sites.^4^ In 2022, NHS Wales, NHS Scotland and Northern Ireland started their own scan for safety programmes.^20^,^22^ In 2023, NHS Supply Chain launched the national Inventory Management and Point of Care Solutions deployment programme in England^23^ to further support up to 20 trusts in implementing inventory systems. In addition, NHS England relaunched its Scan4Safety website.^19^

This paper reports the findings of a study on the experience of implementing Scan4Safety, incorporating GS1 standards, barcoding and related technologies in one demonstrator site.

## Methods

### Setting

The study was conducted in one of the six DHSC Scan4Safety demonstrator sites, a large tertiary acute NHS Trust in the north of England. The DHSC programme required implementation of GS1 standards and barcodes over three use cases – inventory management, traceability of implants and orders/payments to suppliers (online supplemental file 1), leaving hospitals the choice of how and what other use cases to implement. The site extended Scan4Safety to a wider range of use cases and hospital operations, including bed management, infection control, mortuary, estate and security, but excluding medications, and continued implementation after the end of the DHSC programme. This study included all Scan4Safety activities at the study site from the DHSC programme to subsequent developments.

### Research design

The study was exploratory, informed by interpretivist paradigm and theories of healthcare technologies as sociotechnical systems/information infrastructures.^24^ We used a qualitative case study design^25^ with ethnographic methods, taking the hospital as the case. Data were gathered through interviews with hospital staff and document analysis. Data collection took place between October 2023 and May 2024. Study findings are reported in line with the SRQR reporting guideline^26^ (online supplemental file 2).

### Interviews

We conducted semi-structured interviews with staff, asking them to describe and reflect on their experience of implementing or using Scan4Safety systems, and any challenges and benefits they identified (online supplemental file 3). A pragmatic and reflexive approach was undertaken for data sampling,^27^ aiming to interview representatives of the Scan4Safety team, staff responsible for inventory/supply and clinician users of the technology. Interviews started with the Scan4Safety team, who were asked to identify and suggest other participants. This led to interviews with additional stakeholders, at another demonstrator site, GS1 UK and NHS Supply Chain.

Interviews took place online (via MS-Teams). All interviews except one were conducted by two researchers together (VL and AI). With consent, interviews were audio-recorded. Recordings were professionally transcribed, and transcripts checked for accuracy.

### Documents

Documents and other media, including policy documents mentioned by interviewees, reports, videos or websites related to Scan4Safety in general, and specifically about the study site, were obtained. Participants provided organisational Scan4Safety documents in confidence. Other documents were identified by browsing through the Scan4Safety and GS1 websites, searching Google, social media posts and through searching cited references.

### Ethics

The study received ethical approval from an NHS Research Ethics Committee (Ref. 23/NE/0140). The NHS Trust granted research governance approval. Interviewees were informed and gave consent before participating in the study.

### Data analysis methods

Interview transcripts and documents were given a unique identifier (IDn; DOCn); transcripts from interviews with external interviewees were denoted by a suffix of ‘x’ (e.g. ID1-x). Data analysis was inductive. Initial analysis was in parallel to data collection and informed subsequent interview questions. Preliminary findings were discussed with the project advisory group, and their feedback was incorporated into the analysis. The site Medical Director Operations and Scan4Safety Programme Manager (SB, MS) were members of the advisory group and checked the findings for congruence and trustworthiness. Understanding of Scan4Safety was mapped in logic models.

Subsequent analysis involved repeated reading of interview transcripts, seeking answers to the research questions and interpretation through theoretical lenses. Documents were analysed to clarify concepts and events reported in interviews, and to gain a better understanding of the context of implementation. A research assistant helped to extract definitions, benefits and challenges from documents. Two researchers (VL and AI) with a background in information systems in healthcare and other sectors carried out the analysis independently using qualitative data analysis software, NVivo and MS Excel spreadsheets. Themes identified by each researcher were discussed until consensus was reached.

## Results

Eighteen interviews were conducted with 19 participants; one interview was with two participants (table 1). Interviews lasted a mean of ~50 min. Interviews with surgeons took about 10 min. Overall, 67 documents were analysed (online supplemental file 4), including Scan4Safety official guidance, GS1 publications, web posts and videos, policy documents and reports from public inquiries.

**Table 1:** Interviewees’ roles and backgrounds

Internal to case site	Number of participants
Scan4Safety Team - Programme and project management, including cross-trust collaboration and Live Bed State project	5
Procurement and inventory – Senior roles, including also for commercial, e-commerce, specialist supplies and recalls	4
New digital hospital team – assistant role	1
Data science	1
Surgeons	2
Nursing – management role	1
Registered nurse, previously working at case site	1
External to Case Site	
NHS Supply Chains – engagement management	1
Other demonstrator site – programme management	1
GS1 UK – engagement management	2

### Enabling factors

Scan4Safety was considered ‘a pioneering initiative to bring 21st century data standards to our everyday work in the NHS’ (DOC50) - ‘a change of culture’ (ID8) towards data-driven hospitals. Six key enablers for Scan4Safety were identified (online supplemental file 6).

#### Allocated funding

Scan4Safety at the case site was a trust-wide change programme organised in improvement projects, each with specific funding allocation. Its history (online supplemental file 5) is marked by resourcing efforts. Some of these improvement projects after the end of the DHSC programme did not progress mainly due to lack of funding. Participants explained that Scan4Safety could be implemented at relatively low cost by adapting existing systems and technical infrastructure already in place. However, for implementation at scale, and if new information systems must be purchased, available protected funding is indispensable.

#### DHSC involvement, policy and regulations

Suppliers’ adoption of GS1 standards and barcoding of products were prerequisites for the feasibility of Scan4Safety in NHS hospitals. When Scan4Safety began, the largest suppliers of medical devices were already adopting GS1 standards, in response to forthcoming EU and USA regulation. Participants explained that some suppliers appeared to be reticent to make the necessary investment before evidence of use of those standards in the NHS. The DHSC demonstrator programme and a contractual agreement between the NHS and GS1 provided reassurance to suppliers about hospitals' forthcoming use of those standards, facilitated suppliers’ adoption and hence also Scan4Safety implementation.

#### Iterative adaptive development

The programme was developed iteratively, through learning by doing. It used an adaptive, agile and pragmatic user-centred approach— focusing on what was possible, given technical and other constraints. Scan4Safety implementations required constant monitoring, reviewing and maintenance.

#### Clinical executive buy-in and wide stakeholder involvement

Another key enabler of Scan4Safety was a combination of clinical executive buy-in and involving stakeholders. Senior clinicians were involved, able to understand the programme and its benefits, able to communicate those benefits and help unpack foreseeable issues and challenges.

#### Patient focus

All interviewees mentioned that the motivation behind Scan4Safety was improving services for patients, which was also identified as a key issue for clinician adoption. This was recognised by the programme team, leading to a change in the name of the DHSC programme to ‘Scan4Safety’ from a more technical title. This change was believed to facilitate clinicians’ adoption.

Despite Scan4Safety being patient-focused, there was limited involvement of patients in the programme, both at the case site and externally.

#### Linking data

Understanding the importance of combining data and information across hospital operations for the benefit of patients, which an interviewee called ‘*a way of thinking’* (ID1), was essential. The necessary time was invested at the start of the Scan4Safety programme to understand how the GS1 standards could be used and combined. Defining and linking data fields for identification of products, patients, places and processes (‘the four Ps’), and capturing the data, required untangling interconnected hospital processes – each of the four ‘Ps’ as intricate as a *‘spider web’* (ID6x).

### Barriers and complexities of implementation and adoption

Barriers and complexities in the implementation and adoption of Scan4Safety were identified (summarised in table 2, more details in online supplemental file 6), related to both structures and processes across the Scan4Safety life cycle. They were partly interdependent, for example, implementation challenges leading to challenges in adoption. Specifically, in using barcodes for traceability of medical devices, complexities span across the internal and external hospital supply chains, from local and global manufacturers to use at the point of care.

**Table 2:** Summary of barriers and complexities of Scan4Safety in NHS hospitals

Challenges external to the hospital organisation	Challenges internal to the hospital organisation		
	Challenges related to structures	Challenges related to tracking and tracing processes	Challenges related to trade-offs (choices)
IT vendors perceived as not understanding the NHS (business models not fit for the NHS) *** Suppliers of devices not understanding barcodes and GS1 standards *** Suppliers of medical devices delivering directly to clinical areas bypassing standard hospital procurement processes	Data consistency (e.g. use of NHS number not used consistently, product IDs changing over time) *** Data fields for products that do not fit established categories or items with variable states (e.g. breast milk is not a medical product in inventory; beds may be identified as to be cleaned/available/not available) *** Inability to use barcodes (e.g. products/device size, products not set up for scanning) *** Buildings and IT (e.g. availability of devices, quality of Wi-fi, data space, software limitations) *** Staffing (e.g. insufficient staff, not enough time)	Workflow redesign (e.g. having to cater for exceptions, or uncertainties in device use during surgery) *** Porous supply chain and products hidden in hospital wards *** Consistency of barcode scanning (e.g. not done for all devices, all patients, not at the right step in the workflow) *** Risk management (e.g. how to mitigate new risks introduced by the roll-out of barcode scanning, how to foster consistent and correct scanning, how to assess data quality)	Trade-off between time spent scanning vs time spent on tracing items (i.e. not scanning barcodes at the point of supply and use makes tracing difficult and time consuming; tracing items in case of recalls is easier and faster if more time is spent scanning barcodes of items at each point of supply and use) Prioritisation (e.g. where/for which devices should Scan4Safety be implemented?) *** Standardisation vs customisation ^#^ (e.g. identifying what exceptions in workflows to cater for, what workflows should be standardised for best practice, what is best practice, who decides and on what basis?)

Items in this table are interdependent (relate across columns). For example, ‘suppliers bypassing standard hospital processes’ is associated with data challenges (‘devices being supplied without GS1 barcodes’/’inability to use barcodes’) and contributes to challenges to tracking and tracing processes (‘exceptions to workflows’/‘workflow redesign’). # Most of our data for the challenge of standardisation relates to standardisation across NHS trusts, rather than standardisation within a single trust or within the study site.

#### Data-related complexities

Maintaining data quality and establishing confidence in the quality of the data appeared to be a challenge. For example, when GS1 unique identifiers for products changed unexpectedly, or hospital locations changed without informing the Scan4Safety team for a change of identifiers. Most of the devices used for patient care, such as syringes or oxygen tubing, were not supplied to hospitals with GS1 identifiers and barcodes and thus could not be easily tracked. Data challenges were also related to non-standard products and workflows.

Data challenges were addressed in part through attention to work-as-done and redesign of technologies and data processes. For example, historical data were added to the inventory system, in case suppliers changed identifiers for products; processes were adapted to cater for non-standard items that are not already in hospital inventory system.

#### Prioritising and dealing with trade-offs

Part of the challenge was dealing with trade-offs, for example time spent (or saved, by not) tracking items had to be balanced with time saved (spent) tracing recalled items.

There was a perceived cost in staff time taken to scan items. As a consequence, despite Scan4Safety at the study site potentially including ‘*all the moving parts’* (ID2) (all items), in practice, priority had been given to tracking valuable items only – that is, expensive, irreplaceable and associated with safety concerns.

…5% of the products we buy into the Trust that we scan, there’s 95% […] we don’t scan (ID12).

#### Inconsistent and incorrect use of barcoding technologies

The most difficult challenge to address was the lack of consistent clinical engagement – clinicians not scanning barcodes at the point of care or not following appropriate processes when scanning them (eg, scanning after use, instead of before use, thus potentially missing safety alerts). Furthermore, there was a risk of users ‘reverting back’ to old ways of doing their work. This was addressed by repeating user training.

…after a few months it goes back to how it was, there’s a bit of … complacency comes back in again. (ID15)

### Benefits and patient safety risks of Scan4Safety

**Figure 1:**
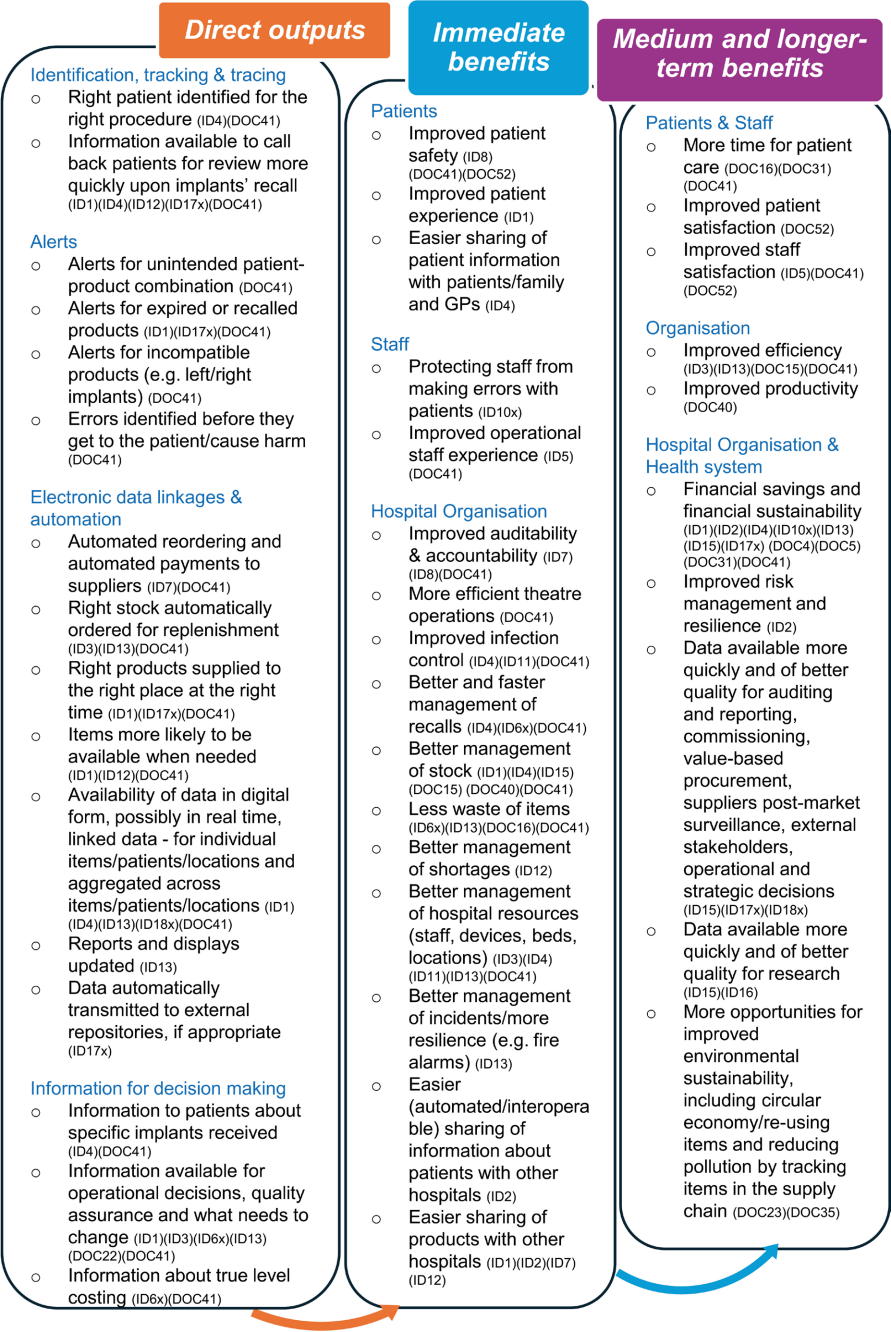
Direct outputs and anticipated benefits of Scan4Safety (IDn indicates interview transcripts; DOCn indicates documents reviewed).

A wide range of anticipated benefits across the varied use-cases of Scan4Safety were identified (figure 1), as well as potential risk of negative outcomes.

#### Potential new risks to patient safety

Two patient safety risks associated with incorrect or inconsistent use of barcoding for patient identification and traceability of implants were identified: 1) patients potentially receiving wrong treatments if their identifying barcode on their wristband is assumed to be correct, when it’s not; 2) data captured through barcodes about the use of implants (usually, but not always, consistently scanned) may be assumed to be complete and accurate when they are not. This could lead to patients with unsafe recalled implants not being called for review.

… If you don’t scan it, where’s the traceability? If you don’t scan it then actually if somebody then says, ‘we never use those, look you can look on the records’, yes you’re right, because we’ve never scanned it, it doesn’t mean we don’t use it. (ID6x)

#### Wide-ranging anticipated benefits

Across both our interviews and review of documents, we identified immediate, medium and longer-term anticipated benefits of Scan4Safety (figure 1) related to individual patient care, groups of patients or the health system. Examples of health system benefits identified were shared inventories for better management of shortages and supplies across organisations (ID2 and ID12), better post-market surveillance of devices (ID18x) and improved interoperability (ID10x). Participants pointed out how benefits from Scan4Safety don’t necessarily manifest ‘*overnight, it takes time to accrue those benefits*’ (ID17x), but benefits are long-lasting (ID10x).

Beyond its outcomes, the process of implementing Scan4Safety was also beneficial—it provided opportunities for learning and *‘wheed(ing) out some really bad practice’* (ID4).

## Discussion

**Figure 2:**
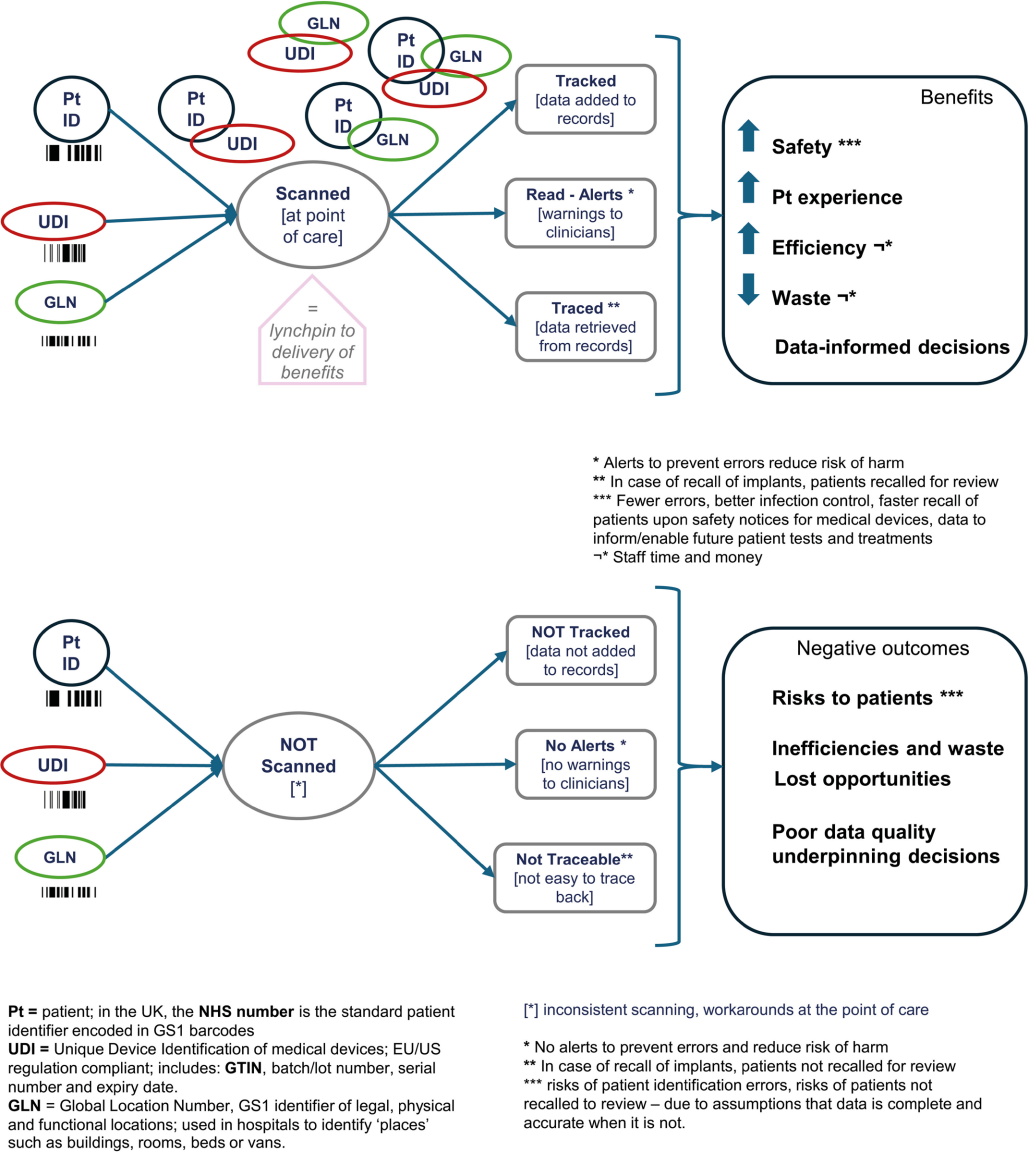
Simplified logic model of Scan4Safety for key use cases: inventory and medical devices – mechanisms and outcomes of scanning (top) and not-scanning (bottom) GS1 barcodes.

Depending on how it is used, the unique identification of people (patients), products and places, captured through barcodes, may deliver a wide range of benefits. The activity of scanning barcodes produces data about items, patients, the time and place they were scanned at, by whom and for what task or procedure. The act of scanning may also generate an alert. The data recorded at the time of scanning may later be used in other activities. The act of scanning is the lynchpin for these capabilities to be in place. We illustrate this in figure 2—a simplified logic model summarising mechanisms and outcomes as identified in this study.

We identified a wide range of anticipated benefits that may be derived from Scan4Safety technologies and GS1 data standards’ adoption, including immediate and longer-term benefits. While US studies of the use of unique identifiers of medical devices (UDIs) have identified a ‘multitude of benefits’^14^ mainly in relation to medical devices, the application at this study site of GS1 standards for the unique identification of locations and people extends the range of benefits to other hospital operational services, such as infection control, or estate and security. We also identified safety risks related to barcoding for patient identification and traceability of implants. These risks are not new and may occur rarely, but it is important that implementers are aware of them and actions taken to mitigate them. Inconsistent scanning of implants might engender an illusion of data completeness, leading to missing patients for review on recall of implants. Hospitals may mitigate this risk through comparison of UDI data in patients records with other hospital data, such as inventory data, possibly applying artificial intelligence methods.^28^

The majority of items in hospital supplies are not tracked—meaning there is scope to expand the Scan4Safety programme to make management of shortages and recalls more effective and efficient.

This study adds nuance to the available evidence about Scan4Safety in NHS hospitals,^4 7 19 29^ highlighting not only benefits but also trade-offs and new safety risks. It enriches the understanding of the safety and efficiency improvements brought by Scan4Safety in the NHS.

The enabling factors of Scan4Safety we identified in this study site confirm those identified in the US context.^14^ Additional factors reported in the literature are expertise in supply chain management and IT,^14^ knowledge and familiarity of UDIs among leadership,^30^ UDI champions^14^ and the awareness of benefits of real-world evidence research drawing on UDI data.^30^ Necessary conditions for implementing Scan4Safety included adequate funding and data infrastructure across a range of systems. Maintaining this infrastructure was found to be a challenge, including dealing with changes in suppliers’ data provision and having clinicians capturing data consistently through barcodes at the point of care. Some of these challenges appear to be related to what could be called ‘data-in-practice’ being different from ‘data-as-imagined’, as well as work-as-done being different from work-as-imagined.^31^

The enabling factors found in this study mirror those reported by the evaluation of another demonstrator programme for whole-scale transformational change and modernisation of healthcare services in London more than a decade ago^27^ - ‘funding, vision, ethos and collective effort’, as well as ‘imaginative and sustained efforts to ensure the long-term sustainability of the various gains’.^27^ Compared with previous modernisation programmes, Scan4Safety has data-specific enablers and challenges, also dependent on external stakeholders (manufacturers/suppliers of medical devices). These data challenges were also reported in studies of the implementation of UDIs in health services in the US.^30^ These studies identified similar internal and external enablers and challenges^30 32^; among key enablers were implementers' appreciation of the infrastructural nature of those data, and the key role of public policy mandates and support; among the challenges was stakeholders’ lack of UDI knowledge. They recommended strategies revolving around education, communication and policy.^32^

The tracking/tracing trade-off involves a redistribution of work between roles, often found in systems implementations that shift administrative (back office) work to clinicians at the point of care.^33^ However, in this site, clinicians were also responsible for back office work (eg, management of recalls/tracing) and the ‘administrative’ act of scanning (tracking) was also capable of generating safety alerts, thus being a constituent part of clinical work, making the issue of redistribution of work between admin/clinical roles more subtle.

Our key recommendations for practice and policy to improve and scale up implementation and adoption of Scan4Safety are in table 3, and questions for further research in online supplemental file 7). The logic model derived from this study provides a framework for the design of evaluations of further Scan4Safety projects.

**Table 3:** Key recommendations for practice and policy

	To facilitate implementation of Scan4Safety
#1	Patients and the public should be involved in design and implementation of Scan4Safety.
#2	For broader implementation of Scan4Safety in the NHS, ensure adequate funding is protected for purpose.
	To facilitate adoption
#3	Extend Scan4Safety to a wider set of items, especially those that may be of low value but essential for patient care delivery.
#4	Scan4Safety implementers are to be responsive to operational work-as-done, rather than only work-as-imagined, and pay attention to data standards-in-practice rather than only data standards-as-imagined.
#5	Scan4Safety systems must be kept up-to-date on an ongoing basis and audited for missing data. Resources must be devoted to this maintenance work.
#6	Scan4Safety training to include clinicians’ education on overall operational processes and data flows beyond those for which they are responsible.

### Strengths and limitations

Strengths of this study are its wide scope over the entire Scan4Safety hospital infrastructure and the modelling of mechanisms and outcomes in a logic model. However, the study was only conducted in one of the six demonstrator sites, meaning that the insights derived from the study may be limited to this one context. Some of the challenges identified may be specific to the Scan4Safety use cases examined. The study focused on past implementations, mainly around the original DHSC programme; we did not observe current Scan4Safety activities. In addition, while we interviewed all members of the Scan4Safety team as well as senior stakeholders, we had limited input from clinicians/users of scanning technologies, who may have had different views and insights into the implementation and benefits of the Scan4Safety programme. The study did not examine the use of GS1 standards and barcoding for medications, as this was not a Scan4Safety use case implemented at the site. There may be specific mechanisms, benefits and issues in scanning medications missing from this study. We did supplement the interview data with analysis of a large number and variety of documentary materials, related to the implementation of the programme. However, this is a sample of all available documents related to Scan4Safety, and by its nature will provide a limited view of the programme, from an organisational and policy perspective. Therefore, some of the nuance related to the ways in which such programmes can be rolled out elsewhere may be missing. Finally, there are limitations with the nature of the interviews, which were conducted at a single point in time, but asked participants to reflect back on the initial implementation of Scan4Safety and progress over time. The interviewees may have emphasised challenges of adoption and engagement still current today over some of the initial challenges they may have encountered at the start, such as in building the IT infrastructure.

## Conclusions

By uniquely identifying patients, products, places and procedures, through data standards, Scan4Safety builds an information infrastructure across hospitals’ internal and external supply chains, with the potential to deliver significant value to patients, clinicians and the NHS.

## Supplementary material

10.1136/bmjhci-2024-101366 online supplemental file 1

10.1136/bmjhci-2024-101366 online supplemental file 2

10.1136/bmjhci-2024-101366 online supplemental file 3

10.1136/bmjhci-2024-101366 online supplemental file 4

10.1136/bmjhci-2024-101366 online supplemental file 5

10.1136/bmjhci-2024-101366 online supplemental file 6

10.1136/bmjhci-2024-101366 online supplemental file 7

## Data Availability

All data relevant to the study are included in the article or uploaded as supplementary information.
