# Supplementary material for: Complexities and capabilities of Scan4Safety in NHS hospitals: a qualitative study of a national demonstrator site
Source: BMJ Health Care Inform. 2026 Jan 14;33(1):e101366. doi: 10.1136/bmjhci-2024-101366 (PMC12815080; doi:10.1136/bmjhci-2024-101366)

Supplementary file 1

Background information about the DHSC Scan4Safety Demonstrator programme and example of linked systems

Demonstrator sites:

- Derby Teaching Hospitals NHS Foundation Trust (now University Hospitals of Derby and Burton NHS Foundation Trust)
- Leeds Teaching Hospitals NHS Trust (LTHT)
- North Tees and Hartlepool NHS Foundation Trust
- Salisbury NHS Foundation Trust
- Royal Cornwall Hospitals NHS Trust
- University Hospitals Plymouth NHS Trust.
- Hull University Teaching Hospitals NHS Trust also independently implemented Scan4Safety at the same time.

The programme required the adoption of GS1 standards for unique identification of products, places and people, and PEPPOL[1] standards for management of invoices and payments in the supply chain, over three use cases. San4Safety GS1 standards and use cases are explained in Box 1.

The examination of the PEPPOL standards was outside the scope of our study.

Further information at: https://www.scan4safety.nhs.uk/

[1] **PEPPOL** = Pan-European Public Procurement Online; a set of specifications for electronic procurement and invoicing; governed by OpenPeppol, a non-profit international association based in Belgium (https://peppol.org/).

Box 1 GS1 standards adopted by the Scan4Safety programme, 'pillars' and core use cases

The GS1 standards:

- **GTIN** = Global Trade Item Number, GS1 identifier of products. Embedded in the **UDI** = Unique Device Identification of medical devices, together with batch/lot number, serial number and expiry date; EU/US regulation compliant.
- **GSRN** = Global Service Relation Number, GS1 identifier of a service relationship between a business and a client; used in the NHS with the NHS number to identify patients.
- **GLN** = Global Location Number, GS1 identifier of legal, physical and functional locations; used in hospitals to identify 'places' such as buildings, rooms, beds or vans.

The pillars:
the core 'building blocks' at the foundation of Scan4Safety - the use of GS1 standards and barcodes for identification of patient, product, place. Once pillars are in place, they can be built on to deliver operational improvements around core use cases.

The core use cases:
three key operational areas the DHSC Scan4Safety programme aimed to deliver improvements on:

1. **Inventory management**: use of GS1 standards for identification of products and locations, to achieve visibility of what is in stock, where, and what is due to expire soon.
2. **Management of product recall and traceability of implants**: use of GS1 standards for identification of products, places and patients, to answers questions about recalled products: whether recalled products are present in the hospital, whether they have been returned; whether they have been used with patients, and if so, which patients.
3. **Purchase to pay – orders and payments to suppliers**: use of GS1 and PEPPOL standards to improve the NHS hospitals business function, with an electronic gateway to automate payments to suppliers.

Implementing Scan4Safety required linking different systems, including, for example:

- Inventory system
- Purchasing system
- Contracting system
- Patient administration system
- Patient record systems
- Theatre scheduling system
- Bed management system
- Location management system
- RTLS / Tracking system

It required some additional hardware such as

- Barcode printers
- Barcode scanners, including tablets (ipads)
- RFID labels and sensors (if RFID included in the implementation).

A visual representation of how systems were linked through the use of GS1 data is provided in the data flow diagram (Figure A). The diagram illustrates flows of the GS1 identifiers for patients, objects and locations between the IT systems, within the hospital supply chain.

Figure A. Example of linked systems in Scan4Safety and data flows

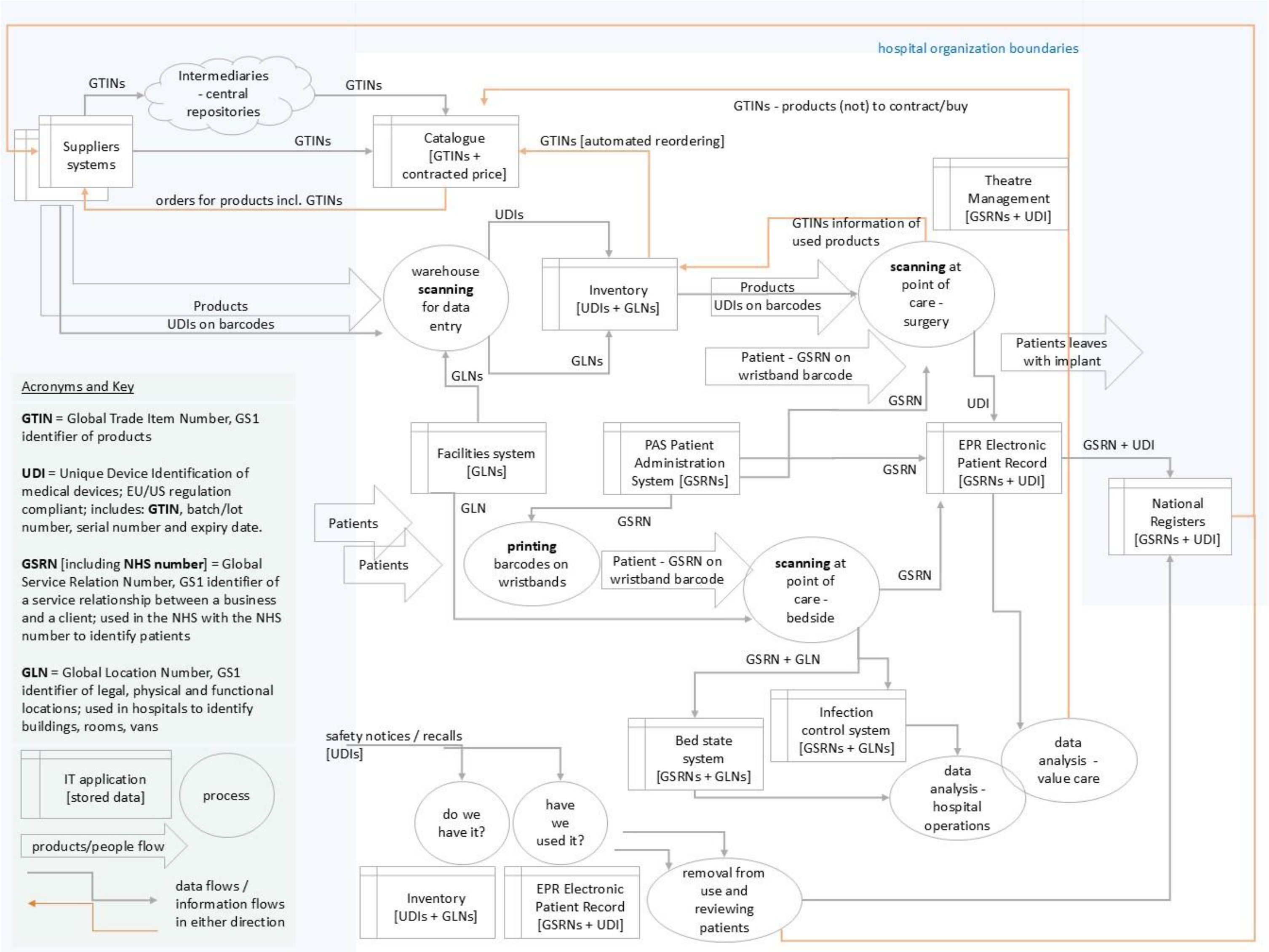

Supplement: online supplemental file 1 [file bmjhci-33-1-s001.pdf]
