# Supplementary material for: Complexities and capabilities of Scan4Safety in NHS hospitals: a qualitative study of a national demonstrator site
Source: BMJ Health Care Inform. 2026 Jan 14;33(1):e101366. doi: 10.1136/bmjhci-2024-101366 (PMC12815080; doi:10.1136/bmjhci-2024-101366)
Supplement: online supplemental file 2 [file bmjhci-33-1-s002.pdf]

Supplementary file

Standards for Reporting Qualitative Research (SRQR)

http://www.equator-network.org/reporting-guidelines/srqr/

Title and abstract

S1. Title - Concise description of the nature and topic of the study Identifying the study as qualitative or indicating the approach (e.g., ethnography, grounded theory) or data collection methods (e.g., interview, focus group) is recommended	Title page - Page 1
S2. Abstract - Summary of key elements of the study using the abstract format of the intended publication; typically includes background, purpose, methods, results, and conclusions	Page 2

Introduction

S3. Problem formulation - Description and significance of the problem/phenomenon studied; review of relevant theory and empirical work; problem statement	Background section
S4. Purpose or research question - Purpose of the study and specific objectives or questions	Background, end of section + Supplementary file 3

Methods

S5. Qualitative approach and research paradigm - Qualitative approach (e.g., ethnography, grounded theory, case study, phenomenology, narrative research) and guiding theory if appropriate; identifying the research paradigm (e.g., postpositivist, constructivist/ interpretivist) is also recommended; rationale[b]	Methods / Research Design section
S6. Researcher characteristics and reflexivity - Researchers' characteristics that may influence the research, including personal attributes, qualifications/experience, relationship with participants, assumptions, and/or presuppositions; potential or actual interaction between researchers' characteristics and the research questions,	Methods / Data analysis section

approach, methods, results, and/or transferability	
S7. Context - Setting/site and salient contextual factors; rationale[b]	Methods / Setting section
S8. Sampling strategy - How and why research participants, documents, or events were selected; criteria for deciding when no further sampling was necessary (e.g., sampling saturation); rationale[b]	Methods / Interviews section
S9. Ethical issues pertaining to human subjects - Documentation of approval by an appropriate ethics review board and participant consent, or explanation for lack thereof; other confidentiality and data security issues	Methods / Ethics section
S10. Data collection methods - Types of data collected; details of data collection procedures including (as appropriate) start and stop dates of data collection and analysis, iterative process, triangulation of sources/methods, and modification of procedures in response to evolving study findings; rationale[b]	Methods / Interviews and Documents sections<br>+ Supplementary files 3 & 4
S11. Data collection instruments and technologies - Description of instruments (e.g., interview guides, questionnaires) and devices (e.g., audio recorders) used for data collection; if/how the instrument(s) changed over the course of the study	Methods / Interviews and Data Analysis sections<br>+ Supplementary file 3
S12. Units of study - Number and relevant characteristics of participants, documents, or events included in the study; level of participation (could be reported in results)	Results section + Table 1<br>+ Supplementary file 4
S13. Data processing - Methods for processing data prior to and during analysis, including transcription, data entry, data management and security, verification of data integrity, data coding, and anonymization/de-identification of excerpts	Methods / Interviews and Data Analysis sections
S14. Data analysis - Process by which inferences, themes, etc., were identified and developed, including the researchers involved in data analysis; usually references a specific paradigm or approach; rationale[b]	Methods / Data Analysis section
S15. Techniques to enhance trustworthiness - Techniques to enhance trustworthiness and credibility of data analysis (e.g., member checking, audit trail, triangulation); rationale[b]	Methods / Data Analysis section

Results/findings

S16. Synthesis and interpretation - Main findings (e.g., interpretations, inferences, and themes); might include development of a theory or model, or integration with prior research or theory	Results section
S17. Links to empirical data - Evidence (e.g., quotes, field notes, text excerpts, photographs) to substantiate analytic findings	Table 2<br>Supplementary file 6<br>+ Quotes reported in Results section/ Barriers and complexities

Discussion

S18. Integration with prior work, implications, transferability, and contribution(s) to the field - Short summary of main findings; explanation of how findings and conclusions connect to, support, elaborate on, or challenge conclusions of earlier scholarship; discussion of scope of application/generalizability; identification of unique contribution(s) to scholarship in a discipline or field	
S19. Limitations - Trustworthiness and limitations of findings	Discussion section/Strengths and limitations

Other

Conflicts of interest - Potential sources of influence or perceived influence on study conduct and conclusions; how these were managed	Declarations section/Competing interests
Funding - Sources of funding and other support; role of funders in data collection, interpretation, and reporting	Declarations section/Funding

[b.] The rationale should briefly discuss the justification for choosing that theory, approach, method, or technique rather than other options available, the assumptions and limitations implicit in those choices, and how those choices influence study conclusions and transferability. As appropriate, the rationale for several items might be discussed together.

Reference:
O'Brien BC, Harris IB, Beckman TJ, Reed DA, Cook DA. Standards for reporting qualitative research: a synthesis of recommendations. Academic Medicine, Vol. 89, No. 9 / Sept 2014 DOI: 10.1097/ACM.0000000000000388
