# Supplementary material for: Complexities and capabilities of Scan4Safety in NHS hospitals: a qualitative study of a national demonstrator site
Source: BMJ Health Care Inform. 2026 Jan 14;33(1):e101366. doi: 10.1136/bmjhci-2024-101366 (PMC12815080; doi:10.1136/bmjhci-2024-101366)
Supplement: online supplemental file 3 [file bmjhci-33-1-s003.pdf]

**Supplementary file 3**

The interview guide addresses the four research questions of our study:

- How was the Scan4Safety programme implemented, and how was it sustained over time?
- What were the enablers of Scan4Safety, and what were the barriers to implementation, if any?
- What benefits have been experienced as a result of Scan4Safety, and by whom and why?
- What are the drawbacks or limitations, if any, of these technologies?

## Interview Guide

Depending on the time available, only a limited set of questions may be possible – these key questions have been signposted (*).

| | |
|---|---|
| Opening | (*) Introductions, confirm consent to recording, remind participant of the aim of the research and answer any questions |
| Background | *Participant role and experience with the Scan4Safety initiative*<br>To start with, could you tell me about your role?<br>(*) Are you/Have you been involved with Scan4Safety in this hospital?<br>What was the participant's role in the Scan4Safety implementation |
| Scan4Safety technologies | *What standards and technologies implemented as part of Scan4Safety/building on the GS1 standards and barcoding*<br>(*) Ask about the history of these implementations: who was involved, when and where were they implemented, what were the expected benefits and challenges, what were the outcomes of these implementations<br>Some of these implementations may be current/in progress. Ask about the current experiences with these implementations<br>Ask about any surprises in the implementation process<br>Solicit examples whenever possible |
| Scan4Safety outcomes: benefits and dis-benefits | *What benefits and drawbacks were experienced from these standards and technologies*<br>(*) Ask about the benefits as experienced specifically in this hospital: what benefits, by whom, how were they reported/measured/documented, were they expected or unexpected |

| | |
|---|---|
| | (*) Ask about the dis-benefits or negative consequences, as experienced specifically in this hospital: what dis-benefits, for whom, how were they reported/measured/documented, were they expected or unexpected<br><br>Solicit examples whenever possible |
| Innovation process: barriers & facilitators | *Perceptions of enablers and constraints to implementation decisions and adoption processes*<br><br>Was there anything that you felt was particularly helpful in making the Scan4Safety initiative [this implementation] possible in this hospital?<br><br>Were there any difficulties or ‘road blocks’ that [may have] delayed or stopped the Scan4Safety initiative [this implementation] in this hospital?<br><br>If so, how and why?<br><br>Solicit examples if possible. |
| Further participants | (*) *Ask if they would recommend others who may have information about Scan4Safety / what was discussed in this interview*<br><br>If so, ask if they wouldn’t mind sending an introduction by email, for us to send an invitation to participate in an interview |
| Closing | (*) Thanks to the participant and ask if they have any questions. |
