# Supplementary material for: Complexities and capabilities of Scan4Safety in NHS hospitals: a qualitative study of a national demonstrator site
Source: BMJ Health Care Inform. 2026 Jan 14;33(1):e101366. doi: 10.1136/bmjhci-2024-101366 (PMC12815080; doi:10.1136/bmjhci-2024-101366)
Supplement: online supplemental file 4 [file bmjhci-33-1-s004.pdf]

Supplementary file 4

Details of documents included in the study.

Row	DOC ID	Author/ Publisher	Year	Title
1.	53	DoH	2007	Coding for success: simple technology for safer patient care
2.	52	DoH	2010	Review of coding for success implementation
3.	49	DoH	2016	GS1 and PEPPOL adoption – Scan4Safety
4.	38	DoH	2014	NHS eProcurement Strategy
5.	59	DoH	2017	Scan4Safety Outline Business Case
6.	61	DoH	2017	Guidance: Scan4Safety implementation requirements - V1.9
7.	39	DHSC	2017	Purchase to pay. A Trust's guide to positive patient identification. V 1.0
8.	1	DHSC	2018	Patient. A Trust's guide to positive patient identification. V 1.0
9.	2	DHSC	2018	Product and catalogue management. A Trust's guide to Product and catelogue management
10.	3	DHSC	2018	Phamacy. A Trust's guide to positive patient identification. V 1.0
11.	46	DHSC	2017	Place. A Trust's guide to positive patient identification. V 1.0
12.	56	NHS Hull University Teaching Hospitals	2022	How to implement RFID (Radio Frequency Identification) and RTLS. (Real Time Locating System) from planning to performing
13.	57	WYAAT	2022	West Yorkshire Association of Acute Trusts Annual Report 2021/2022
14.	58	WYATT	nd	Scan4Safety Governance Diagrams _v1.4
15.	12	DHSC	2023	The government's 2023 mandate to NHS England
16.	60	NHS Supply Chain	2023	In-Trust Inventory Management Programme - Customer Information Pack
17.	24	Scott Pryde/NHSE	2024	The outcomes and registries programme: context and key priorities
18.	35	NHS England	2024	Delivering a ‘Net Zero’. National Health Service
19.	14	DHSC	2024	The 2024 Budget and NHS productivity: The Health and Social Care Secretary, Victoria Atkins, spoke at the Nuffield Trust Summit about the Budget and NHS productivity.
20.	6	Baroness Julia Cumberlege	2019	The Independent Medicines and Medical Devices Safety Review
21.	21	HSSIB	2024	Positive patient identification
22.	34	Thirlwall Inquiry Legal Team	2024	Review of implementation of recommendations from previous inquiries into healthcare issues prepared by the Thirlwall Inquiry Legal Team
23.	44	The Health Foundation	2022	Evaluation of NHS partnership with Virginia Mason Institute
24.	33	FDA	2024	FDA GUDI Database guidance
25.	36	European Commission	2020	Unique device identification (UDI) system: under the EU medical devices regulations
26.	37	European Commission	nd	Medical devices - EUDAMED
27.	60	NHS Supply Chain	2023	In-Trust Inventory Management Programme - Customer Information Pack

Row	DOC ID	Author/ Publisher	Year	Title
28.	11	NHS Supply Chain	2023	In-Trust Inventory Management Systems (IMS)<br>How we are deploying inventory management capability into acute hospitals
29.	25	NHS Supply Chain	2024	Data standards - supplier product coding policy. NHS Supply Chain
30.	26	Frankie Wallace	2024	NHS Supply chain launches policy on data standards for supplier product coding
31.	28	NAO	2024	NHS Supply chain and efficiencies in procurement
32.	51	GS1 UK	2015	GS1 UK: Making a difference in healthcare
33.	40	GS1 and International Hospital Federation (IHF)	2019	Global identifiers for enhancing efficiency and patient safety
34.	41	GS1 UK	nd	A scan of the benefits: the Scan4Safety evidence report: Improving patient safety and saving money using point-of-care scanning in the NHS
35.	42-1	GS1 UK	2019	GS1 Healthcare Reference Book<br>2021-2022 Stories of successful implementations of GS1 standards
36.	42-2	GS1 UK	2020	GS1 Healthcare Reference Book<br>2021-2022 Stories of successful implementations of GS1 standards
37.	50	GS1 UK	2020	New report proves point-of-care scanning in the NHS improves patient safety, releases back clinical time to care and saves the NHS millions of pounds
38.	45	Chris Florey/GS1 UK	2020	GS1 UK Location Manager: The National GLN Registry for Healthcare Suppliers and NHS Trusts
39.	7	GS1 UK Healthcare User Group	2021	Inventory management systems guidance
40.	8	GS1 UK Healthcare User Group	2021	Surgical instrument traceability guidance
41.	9	GS1 UK Healthcare User Group	2022	Asset management using GS1 standards
42.	15	Simon Hemingway/GS 1 UK	2023	The Government's mandate for the NHS to adopt barcode technology is positive - but is the NHS ready?
43.	43	GS1 UK	2024	How can GS1 Standards be used to better manage patient flow
44.	55	GS1 Healthcare	2024	United Kingdom: end-to-end product traceability enables safer, more efficient care for Lancashire
45.	18	GS1 UK	2024	Barcode verification for hospitals<br>Submit a product barcode for verification
46.	23	GS1 UK	2024	Reducing the global impact of environmentally harmful anaesthetic gases using a medical device
47.	29	GS1 UK	2024	NHS England reinforces drive for point of care scanning with launch of new Scan4Safety website
48.	16	GS1 Healthcare	2024	Scan4Safety: Giving time back to patient care at Dublin's Tallaght University Hospital (TUH)
49.	13	European association of hospital pharmacists	2023	Autonomous pharmacy framework beta version: Governance of data and interoperability

Row	DOC ID	Author/ Publisher	Year	Title
50.	19	European association of hospital pharmacists	2024	Autonomous pharmacy framework beta version: Workforce Allocation
51.	20	European association of hospital pharmacists	2024	Autonomous pharmacy framework beta version: Project Management
52.	62	NHS England	2024	Scan4Safety website: https://scan4safety.nhs.uk/
53.	17	NHS	2024	First steps for NHS Trusts. https://scan4safety.nhs.uk/how-to-get-started/first-steps-for-nhs-trusts/
54.	30	NHS	2024	The Scan4Safety website has a fresh new look! https://scan4safety.nhs.uk/the-scan4safety-website-has-a-fresh-new-look/
55.	31	NHS	2024	Evidence [Scan4Safety webpage] https://scan4safety.nhs.uk/evidence/
56.	54	Salisbury NHS Foundation Trust	2016	Salisbury NHS Foundation Trust - Introducing Scan4Safety [YouTube recording] https://www.youtube.com/watch?v=_6nlSDGkB4c
57.	47	Jon Hoeksma	2017	NHS barcode project aimed at improving patient safety. Digital Health https://www.digitalhealth.net/2017/01/nhs-barcode-project-aimed-at-improving-patient-safety/
58.	5	Owen Hughes	2018	Leeds credits 'dramatic' improvement in patient visibility to Scan4Safety. Digital Health https://www.digitalhealth.net/2018/02/leeds-credits-dramatic-improvement-scan4safety/
59.	48	Laura Stevens	2017	Leeds Teaching to pull data from barcodes straight into EPR. Digital Health https://www.digitalhealth.net/2017/09/leeds-teaching-data-from-barcodes-into-epr/
60.	64	GS1 UK, with LTHT	2021	How GS1 and PEPPOL standards enable traceability in the clinical setting [YouTube recording] https://www.youtube.com/watch?v=aTFxTE_fzt0
61.	10	Andrea Chipman	2023	Govt mandate to NHSE calls for trusts to adopt barcode scanning by 2024. Digital Health https://www.digitalhealth.net/2023/07/govt-mandate-to-nhse-calls-for-trusts-to-adopt-barcode-scanning-by-2024/
62.	32	Stephen Bush	2023	Live bed state: reducing the strain on wards and staff at Leeds Teaching Hospitals, Health Services Journal https://www.hsj.co.uk/technology-and-innovation/live-bed-state-reducing-the-strain-on-wards-and-staff-at-leeds-teaching-hospitals/7035334.article
63.	27	GS1 webinar, with Simon White, NHS Scotland National Programme Director	2024	Taking a National approach to scanning across Scotland [YouTube recording] https://www.youtube.com/watch?v=cm99jkNbwfw
64.	22	The Leeds Teaching Hospital Trust	2024	Using barcodes to power Live Bed State in the NHS GS1 UK Healthcare https://twitter.com/gs1uk_hc/status/1778079331606028322?s=61

Row	DOC ID	Author/ Publisher	Year	Title
65.	63	GS1 UK	2024	GS1 UK Scan4Safety and UDI forum, with presentations from Anna Stec, senior project manager, Scan4Safety England, NHS England, and Frankie Wallace, data standards engagement manager, NHS Supply Chain, Mark Songhurst, Scan4Safety, programme manager, LTH NHS Trust, Simon White, programme director, Scan for Safety Scotland, NHS NSS and Andy Smallwood, assistant director of procurement and SfS lead, NWSSP (Wales), among others. https://www.gs1uk.org/sites/default/files/Scan4Safety_and_UDI_forum_slides_July24.pdf
66.	65	GS1 US	2024	The Barcode Dilemma: Uncovering Challenges and Opportunities to Increase Hospital Barcode Scanning for Enhanced Visibility. GS1 US https://www.gs1us.org/articles/the-barcode-dilemma
67.	66	Patient Safety Learning, with NHS Blood & Transplant	2024	Using barcode scanning technology to improve blood group testing in unborn babies https://www.pslhub.org/learn/commissioning-service-provision-and-innovation-in-health-and-care/digital-health-and-care-service-provision/using-barcode-scanning-technology-to-improve-blood-group-testing-in-unborn-babies-r12081/
