# Supplementary material for: Complexities and capabilities of Scan4Safety in NHS hospitals: a qualitative study of a national demonstrator site
Source: BMJ Health Care Inform. 2026 Jan 14;33(1):e101366. doi: 10.1136/bmjhci-2024-101366 (PMC12815080; doi:10.1136/bmjhci-2024-101366)
Supplement: online supplemental file 5 [file bmjhci-33-1-s005.pdf]

Supplementary file

A timeline of Scan4Safety at the study site and related policy.

Right column: events internal to study site; Left column: key external events and policy documents.

Key external events and policy documents	Year	Events internal to study site
PIP Breast Implant identified as dangerous[1]	2011	8+ months to identify and recall patients with PIP breast implants
DoH (now DHSC) **e-procurement strategy**[2]	2014	
GS1 & PEPPOL Demonstration Programme launched: 6 demonstrator sites awarded funding	2015	Site awarded funding as a demonstrator site
Programme renamed Scan4Safety<br>**Lord Carter Review**[3]	2016	Site receives DHSC funding and begins implementation
Demonstrator sites implementation work		Site implementation of Scan4Safety pillars and core use cases
EU medical device regulation[4]		
	2018	End of DHSC funding (maintenance of Scan4Safety ongoing, business as usual)
DHSC audit of demonstrator sites –benefits confirmed - awarding of GS1 certifications	2019	Site Scan4Safety certification
**Cumberlege review** on harm of devices and medications – launched 2018, published 2020[5] – recommends register of implantable devices		
COVID-19 pandemic begins<br>NHS-wide issues with procurement and distribution of	2020	Site awarded funding to implement a shared

[1] NHS.uk. (2022). *PIP breast implants*. https://www.nhs.uk/conditions/pip-implants

[2] Finance & NHS Directorate, Procurement, Investment & Commercial Division (PICD) (2014) NHS eProcurement Strategy, Department of Health: https://assets.publishing.service.gov.uk/government/uploads/system/uploads/attachment_data/file/344574/NHS_eProcurement_Strategy.pdf

[3] Operational productivity and performance in English NHS acute hospitals: unwarranted variations https://www.gov.uk/government/publications/productivity-in-nhs-hospitals (2016)

[4] Regulation (EU) 2017/745: https://www.legislation.gov.uk/eur/2017/745/contents

[5] First Do No Harm – The report of the Independent Medicines and Medical Devices Safety, chaired by Baroness Julia Cumberlege: https://www.immdsreview.org.uk/Report.html

scarce medical supplies (e.g. ventilators)		catalogue/inventory system with other local trusts
EU medical device regulation coming into force in EU countries and Northern Ireland	2021	Site continues development of core and other use cases
NHS England Outcomes and Registries Programme established[6]	2022	
NHS Supply Chain In-Trust Inventory Management Systems programme launched<br>NHS England Federated Data Platform[7] launched	2023	Plans for RFID in ED at study site
NAO report on state of NHS supply[8]<br>HSSIB National learning report Positive patient identification[9]<br>forthcoming new regulation for medical devices in the UK[10]	2024	…development of other use cases ongoing.

[6] NHS England. The NHS England Outcomes and Registries Programme, established in 2022 to develop a single, unified registry solution – the Outcome Registries Platform, consolidating existing registries, to address data and vigilance gaps: https://www.england.nhs.uk/outcomes-and-registries-programme/

[7] NHS England (2024) NHS Federated Data Platform Programme Engagement Portal: https://fdp.england.nhs.uk/home

[8] NAO (2024) NHS Supply Chain and efficiencies in procurement, January 2024

[9] HSSBI (2024) National learning report - Positive patient identification, February 2024: https://www.hssib.org.uk/patient-safety-investigations/positive-patient-identification/national-learning-report/

[10] UK Government (2024) Standard - Implementation of the future regulations - Updated 3 July 2024: https://www.gov.uk/government/publications/implementation-of-the-future-regulation-of-medical-devices/implementation-of-the-future-regulations
