# Supplementary material for: Complexities and capabilities of Scan4Safety in NHS hospitals: a qualitative study of a national demonstrator site
Source: BMJ Health Care Inform. 2026 Jan 14;33(1):e101366. doi: 10.1136/bmjhci-2024-101366 (PMC12815080; doi:10.1136/bmjhci-2024-101366)
Supplement: online supplemental file 6 [file bmjhci-33-1-s006.pdf]

Supplementary file

Table i. Key enablers of Scan4Safety implementation

Note: Interview data identified by a number (e.g. ID1), with 'x' signposting interviewees external to the case site. Documents are identified by a number (e.g. DOC1).

Enabler	Example from the data
Funding availability	*…it helped that […] we did have some pump priming - so funding for us was beneficial. […] would we have done it without that funding? Maybe not actually, back in the day. [ID1]*<br><br>*…because we've had funding we've been able to look at this holistically across the hospital and tackle some real challenges. [ID4]*<br><br>*I think there's a lot down to funding. […] the difficulty is getting Trusts to do anything upfront without some funding. [ID17x]*
DHSC involvement and regulation	*… getting those suppliers on board [in using GTINs] […] All of a sudden, the Department of Health came and said, "Everyone's doing this." There were lots of demonstrator days, lots of talking to the suppliers, … [ID7]*
Clinical executive buy-in and wide stakeholder involvement	*…senior support clinically is absolutely vital. [ID10x]*<br><br>*… operational buy-in. It's very senior leadership, so exec level leadership and clinical leadership were probably the two biggest things to enabling [Scan4Safety] […] [also] We've always had IT engagement as well as other operational colleagues, so nursing, even estates and facilities at the start, […] very much working with those stakeholders at an operational level. [...] the biggest factors for success, and we've continued to do that and are very good at doing that. [ID1]*
Patient focus	*… we always try and think about it from the patients' point of view first, what benefits does this give to them? Does this give them a better outcome? […] And then how do we use the data and technology to support and try and continue to improve that? [ID1]*<br><br>*… a real patient focus to everything that we developed. [ID4]*
Agile incremental, iterative, adaptive approach	*…some of it came as we went. So some of it we also developed as we went in conversation. [ID4]*<br><br>*…We continue to make changes and get the product, get the standard in - those GS1 standards, your GTINs, your GLNs, and then you can, as you go down the journey, you continually look at the improvements you can make. [ID7]*<br><br>*… [technologies] get smarter and smaller and easier to work with. So, things change. We constantly need to look at what is available now, what can we do with that now? [ID1]*
Data literacy – understanding linked data and systems	*…We spent time understanding the standards and how we can combine them to get benefits [extract from transcript of video recorded presentation [DOC64]*<br><br>*A lot of people think Scan4Safety is a single system. A lot of people think it is an inventory management system. It's not, it's a way of thinking more than anything. […] It's looking at patient, place, product, and procedure and most of that stuff, you're already capturing in some ways, it is just about how you try and link it together …[ID1]*

Table ii. (Extended Table 2). Summary of the barriers and complexities of Scan4Safety in NHS hospitals – with examples from the data

Note: Items in this table are interdependent (relate across columns). For example, 'suppliers bypassing standard hospital processes' is associated with data challenges ('devices being supplied without GS1 barcodes'/'inability to use barcodes') and contributes to challenges to tracking and tracing processes ('exceptions to workflows'/'workflow redesign'). # Most of our data for the challenge of standardisation relates to standardisation across NHS trusts, rather than standardisation within a single trust or within the study site.

**Challenges external to the hospital organisation** (not under hospital management control)		**Challenges internal to the hospital organization**					
	**Data examples**	Challenges related to **structures**	**Data examples**	Challenges related to **tracking and tracing processes**	**Data examples**	Challenges related to **trade-offs (choices)**	**Data examples**
IT vendors perceived as not understanding the NHS (business models not fit for the NHS)<br>***<br>Suppliers of devices not understanding barcodes and GS1 standards<br>***<br>Suppliers of medical devices delivering directly to clinical areas bypassing standard hospital procurement processes	*…suppliers of solutions not understanding the NHS. […] One of the big things is subscription funding is all well and good if you've got ten people in the department. […] [for a hospital] an enormous cost. And also then you become beholden to one supplier. [ID4]*<br>***<br>*…our big challenge is suppliers. So suppliers not understanding barcodes, …[ID4]*<br>***<br>*Some reps, it happens all the time where they may want a clinician to trial a product that we haven't contracted for yet… [ID7]*<br><br>*…if it hasn't come in via a fully compliant route, we don't know it's in the Trust, so therefore we can't account for it […] this doesn't happen very often but it does happen. And the non-compliant routes are that […], it's come in already inside a patient from another Trust, […] The other way is that companies deliver products directly… [ID12]*	Data consistency (e.g. use of NHS number not used consistently, product IDs changing over time)<br>***<br>Data fields for products that do not fit established categories or items with variable states (e.g. breast milk is not a medical product in inventory; beds may be identified as to be cleaned/available/not available)<br>***<br>Inability to use barcodes (e.g. products/device size, products not set-up for scanning)	*…the data that is within our control, the patient data, the place data, the procedure data, not really so much of an issue. The odd thing here and there, but very, very minor and it's usually fixed in, you know, within a few minutes. But product data is especially difficult. [ID6x]*<br>***<br>*… [breast milk] it is difficult as to where it fits. It's not quite a medical product, so it's not a medicine that sits in there, it's not quite a product that we give… [ID4]*<br>***<br>*…One of the main big things being like the size of the tags, so they are - for an active system they're quite big. So, if you wanted to put that on like a smaller pump, half of your pump's already taken up just from where you stick onto your pump type thing. So, you know, there are some challenges around that. [ID5]*<br><br>*..in theory all products come with a barcode, but they don't; so you know, kind of that is a massive issue to us; or they put one on the outer box […]. When you get two-hundred, four-hundred, a thousand to a box, they often put them on the*	Workflow redesign (e.g. having to cater for exceptions, or uncertainties in device use during surgery).<br>***<br>Porous supply chain and products hidden in hospital wards.	*… try to do it the other way and almost reverse that because we do get in what we call loan kits […] don't have within the IMS [inventory system], so you want to scan that product as gone to the patient, but we're reversing it… [ID7]*<br><br>*…sometimes you're not sure what sort of product you're going to be using, exactly […] You might have two or three options and you might decide at the last minute to use option three rather than option one. But for many operations you'll have a very good idea before you start operating what you're going to use. So you could scan it at the very start of the operation, on the operation day, and check for date of issue and date of expiry at that point, that would be a very useful thing to do… [ID17].*<br>***<br>*…I have hidden stuff at the back of the laundry room because I might need it and I've experienced not having it and what impact that has had, so I'll hide it in case I need it. That doesn't do us any good. I now see a bigger picture and I get very frustrated when*	Trade off between time spent vs time spent on tracing items<br>(i.e. not scanning barcodes at the point of supply and use makes tracing difficult and time consuming;<br>tracing items in case of recalls is easier and faster if more time is spent scanning barcodes of items at each point of supply and use).<br><br>Prioritisation (e.g. where/for which devices should Scan4Safety be implemented?)<br>***<br>Standardisation versus customisation #<br>(e.g. identifying what exceptions in workflows to cater for, what workflows should be standardised for best practice, what is best practice, who decides and on what basis?)	*…would be very labour intensive. … you're then asking your clinical staff … If I'm caring for a patient in a twelve-hour shift, I might have …one hundred plus different products I'm having to scan on top of the fact I'm really really busy, there's not enough staff on the wards […] There is so much more scope and … It's like stepping very gradually. And it's balancing. What is expensive that we need to track, and I get that, I do get that, and what is patient risk and what's supply. [ID12]*<br>***<br>*…the intention from the start was always to standardise. That was always our remit right from the beginning, so it was always about – […] finding best practice regardless of where that was. And that is why we had people travel between sites and why we had that collaboration […] The intent was always to understand and capture what was happening and the variants between it and why, and then look at: where is the best practice there? [ID2]*<br><br>*…they're sitting back going, we don't want*

**Challenges external to the hospital organisation** (not under hospital management control)			**Challenges internal to the hospital organization**					
	**Data examples**		Challenges related to **structures**	**Data examples**	Challenges related to **tracking and tracing processes**	**Data examples**	Challenges related to **trade-offs (choices)**	**Data examples**
			<br>Buildings and IT (e.g. availability of devices, quality of Wi-fi, data space, software limitations)<br><br><br>Staffing (e.g. insufficient staff, not enough time).	*outside of the box, but of course we don't take that box into us, we don't carry a great big box to take three, you know, three sample vials through, we carry the three sample vials through separately. So not everything has their own barcode and that is a problem... [ID6x]*<br><br>***<br>*…I think the system they've got at the moment, say a pump's tagged. It could show you that it's actually on the floor above to the room that it's in because it's not quite accurate enough to show you exactly where in the building it is. [ID5]*<br><br>*IT can be a roadblock and that is not necessarily IT themselves. It still needs investment. So, data takes up space and […] just from a resource perspective, those can be difficult to overcome and undertake, again, time to resolve. [ID1]*<br><br>***<br>*…today is kind of a classic example. So if you have a shortfall of nurses, for example, on a ward, and […], there's a lot of work behind getting a patient freed up and bed ready and things… [ID13]*	***<br>Consistency of barcode scanning (e.g. not done for all devices, all patients, not at the right step in the workflow).<br><br>***<br>Risk management (e.g. How to mitigate new risks introduced by the roll-out of barcodes scanning, how to foster consistent and correct scanning, how to assess data quality)	*people do that, but I understand why they do it because I've done it. [ID12]*<br><br>***<br>*…where people don't follow process, […] they do not think that if they don't [scan], you know, they expect everybody to be mind-readers. They know what they've used, we don't […] People need to be, need to follow a process, understand why we follow a process. […] I know […] there's odd ones in there that haven't been scanned... [ID15]*<br><br>*…you've also got to make sure that the education and the training that goes into it is absolutely spot on. Because if it's not, the compliance [with scanning barcodes] is going to be really low and people are not going to scan. [ID10x]*<br><br>***<br>*…at any one point she needs to be able to see what she's got with confidence that transactions have happened and that that is what we've got, […] and she can match that with what we've got coming in […] A handful of people doing the right thing - if the people who are doing it all the time aren't doing it right. So [compliance] is key [ID13]*		*things done [as in other trust] ... [ID-a]*
