# Supplementary material for: Complexities and capabilities of Scan4Safety in NHS hospitals: a qualitative study of a national demonstrator site
Source: BMJ Health Care Inform. 2026 Jan 14;33(1):e101366. doi: 10.1136/bmjhci-2024-101366 (PMC12815080; doi:10.1136/bmjhci-2024-101366)
Supplement: online supplemental file 7 [file bmjhci-33-1-s007.pdf]

Supplementary material

Questions for further Scan4Safety research

We outline questions for further research to contribute to implementation, adoption and evaluation of Scan4safety.

Scan4Safety research must be inclusive of all stakeholders and may have to span across both the internal and external supply chains of a hospital organisation.

Further research to inform implementation

1. Research on patients' experiences and views of the value of Scan4Safety

The motivation behind Scan4Safety is improving services for patients, but little is known about patients' views and experiences of Scan4Safety. Questions remain, that **should** be answered by research, about:

- What are patients' views about Scan4Safety?
- What are their hospital experiences of identification of their person, their care and their records?
- Are there issues of equity and inclusion in the identification of their person, their care and their records, by the use of barcodes?
- What are their priorities for application of Scan4Safety methods/principles, and to which use case(s)?

2. Research for evidence of operational issues prior to using Scan4Safety

Scan4Safety is expected to improve safety and efficiency of services. However, evidence of the issues to be addressed and improved with Scan4Safety appears to be limited. In particular:

- Patient identification errors are reported in patient safety incidents reports. Which of these errors may have been addressed by barcode scanning of patients' wristbands? Are there specific areas or aspects of patient care that may be particularly prone to patient identification errors, and where the use of standard unique identifiers and barcodes may reduce the risk of these errors occurring?

- There is extensive evidence of medication errors across the entire hospital medication process. BCMA (Bar-coded medication administration) is implemented to reduce administration errors. Are there other stages in the medication process where the scanning of barcodes may reduce the risk of errors? Which type of error may be addressed by barcode scanning of medications?

- There is a lack of evidence of operational issues that may be addressed by tracking patients' and devices' locations across a hospital, especially in the emergency department. This lack of evidence may contribute to an underappreciation of the scale of operational inefficiencies traditionally experienced in NHS hospitals. Further research should gather evidence in organizations that *do not* track patients' locations and locations of devices, for: clinicians' time spent looking for patients or devices, patient 'lost' in the system, patients leaving without being seen, patients' harm and extra waiting time due to clinicians' difficulties locating medical devices.

- Not enough is known of the work involved in the management of safety recalls in hospitals and the safety risks associated with recalls of implants. Research should gather evidence on the time spent by clinical and other staff on tracing recalled items in the hospital, and whether any patients have been missed and not called for review after a safety notice and recall of implants.

- In theatre, operational and safety issues may be addressed by barcoding implants, scanning implants at the point of care, and sharing implants' identifiers with patients. There is a lack of evidence of risks and inefficiencies caused by not implementing Scan4Safety in these areas, which may contribute to an underappreciation of its benefits. Further research should gather evidence in organizations that *do not* scan implants, and with patients who do not have information about their implants, for example: surgeries being cancelled or postponed for lack of available implants, patients having treatments or scans cancelled or postponed for lack of information on implants they may have (e.g. incompatible with MRI scans).

Evidence of operational issues that may be addressed by barcode scanning would facilitate hospitals drawing business cases for future Scan4Safety investments. Research in these areas would also provide baseline evidence for evaluation of benefits after new implementations.

3. Research for evidence of safety benefits of Scan4Safety

Benefits of Scan4Safety have been demonstrated by the DHSC demonstrator programme. However, these appeared to be limited to financial and time benefits of the implementation. We have identified a range of benefits, but quantifying these benefits was outside the scope of our study. Research should quantify the safety benefits of Scan4Safety, in particular in terms of reduction of incidents and harm, across the specific use cases of Scan4Safety. This research should comprehensively assess the benefits, including both direct and indirect benefits, and immediate and medium-longer term benefits.

Evidence of safety benefits should facilitate hospitals drawing business cases for future Scan4Safety implementations.

Further research to inform adoption

4. Research on sociotechnical factors facilitating or hindering scanning barcodes at the point of care

Human factors-informed research has identified sociotechnical barriers and facilitators of scanning barcodes in hospitals, identifying structural factors such as sufficient number of devices, availability of Wi-Fi, good quality printing, and quality of light as facilitators of adoption, for example. Additional factors may be related to implementation processes, such as insufficient training, inconsistent guidance, exceptions to scanning rules. However, most of this research has been done on scanning of medications (e.g. with BCMA – barcoded medication administration systems), while less is known about barriers and facilitators to adoption of barcodes scanning in other use cases, such as for traceability of implants. Specific contextual factors may be at play across the different use cases.

Organizations implementing Scan4Safety must carry out this type of research as part of their analysis of the work-as-done at the point of care so that specific issues may be resolved before roll-out.

5. Research on clinicians' views and experiences of the value of scanning barcodes

Research on the challenges of scanning medications at the time of administration (with BCMA) suggests behavioural factors may also contribute to clinicians not scanning barcodes at the point of care. These factors are likely to be related to local safety culture and clinicians' perceptions about usefulness of barcode scanning, professional identity and values. Our interviewees expressed these issues in terms of questions such as 'what's in it for me'? [ID11] and 'what does it mean to them when they're asked to start scanning?' [ID12]. Gathering evidence into these issues requires research sensitive to culture and values, such as those informed by anthropology and ethnography.

- Research should be carried out on clinicians' perceptions of value of scanning barcodes at the point of care and the role local culture may play in informing those perceptions and subsequent behaviours.
- Research may also be carried out on how best to communicate meaning and value of Scan4Safety to clinicians – whether as part of training or information campaigns.

6. Research on how to mitigate new safety risks

New technology implementations may have unintended negative consequences and create new risks. We identified new risks to patient safety associated with staff or clinicians' potentially assuming that barcodes identifying patients are correct, when they are not, or that data captured in the system about implanted medical devices are accurate and complete, when they are not. There may be other new risks we

have not identified. Further research should confirm the significance of these risks, and what actions can be taken to mitigate them.

Further research to inform evaluation

7. Metrics and methods for evaluation of Scan4Safety

Research should be done on what would constitute meaningful evaluations of Scan4Safety, for the specific hospitals implementing it and for the wider NHS.

More specifically, questions may be asked on what issues and benefits may be measured (and how) when implementing Scan4Safety at scale, across the NHS in England and across the four UK nations.

- Are there specific benefits that may emerge at a wider system level, beyond the aggregation of benefits derived by single organizations?
- Are there any unexpected negative consequences at a wider system level?

Are there questions of equity when Scan4Safety is *not consistently* implemented *across all* NHS hospitals?
